# Dendritic Cell-Mediated Phagocytosis but Not Immune Activation Is Enhanced by Plasmin

**DOI:** 10.1371/journal.pone.0131216

**Published:** 2015-07-01

**Authors:** Rachael J. Borg, Andre L. Samson, Amanda E.-L. Au, Anja Scholzen, Martina Fuchsberger, Ying Y. Kong, Roxann Freeman, Nicole A. Mifsud, Magdalena Plebanski, Robert L. Medcalf

**Affiliations:** 1 Australian Centre for Blood Diseases, Monash University, Alfred Medical Research and Education Precinct, Melbourne, 3004, Victoria Australia; 2 Department of Biochemistry and Molecular Biology, Monash University, Clayton, 3181, Australia; 3 Department of Immunology, Monash University, Alfred Medical Research and Education Precinct, Melbourne, 3004, Victoria, Australia; 4 Department of Medicine, Monash University, Alfred Medical Research and Education Precinct, Melbourne, 3004, Victoria, Australia; 5 Department of Allergy, Immunology and Respiratory Medicine, Alfred Medical Research and Education Precinct, Melbourne, 3004, Victoria, Australia; University of California San Francisco, UNITED STATES

## Abstract

Removal of dead cells in the absence of concomitant immune stimulation is essential for tissue homeostasis. We recently identified an injury-induced protein misfolding event that orchestrates the plasmin-dependent proteolytic degradation of necrotic cells. As impaired clearance of dead cells by the innate immune system predisposes to autoimmunity, we determined whether plasmin could influence endocytosis and immune cell stimulation by dendritic cells – a critical cell that links the innate and adaptive immune systems. We find that plasmin generated on the surface of necrotic cells enhances their phagocytic removal by human monocyte-derived dendritic cells. Plasmin also promoted phagocytosis of protease-resistant microparticles by diverse mouse dendritic cell sub-types both *in vitro* and *in vivo*. Together with an increased phagocytic capacity, plasmin-treated dendritic cells maintain an immature phenotype, exhibit reduced migration to lymph nodes, increase their expression/release of the immunosuppressive cytokine TGF-β, and lose their capacity to mount an allogeneic response. Collectively, our findings support a novel role for plasmin formed on dead cells and other phagocytic targets in maintaining tissue homeostasis by increasing the phagocytic function of dendritic cells while simultaneously decreasing their immunostimulatory capacity consistent with producing an immunosuppressive state.

## Introduction

Following tissue injury efficient mechanisms mediate the recognition and removal of dead cells [1,2] in a process that minimises exposure to toxic and immunogenic intracellular epitopes. Conversely, defects in dead cell removal underlie many diseases including lupus [3], cystic fibrosis [4], atherosclerosis [5] and bacterial infection [6]. Dead cells facilitate their innocuous removal via the presentation of signals that engage pro-degradation enzymes and promote phagocytic clearance. These signals, called Damage-Associated Molecular Patterns (DAMPs), represent an array of generic motifs that are recognised by a cognate set of humoral factors and peri-cellular receptors which, in turn, instruct the efficient removal of dead cells [1,2]. DAMPs are also critical in influencing downstream responses to injury, such as inflammation, immune tolerance and repair [1,2].

We recently demonstrated that tissue necrosis triggered the misfolding and aggregation of intracellular proteins [7,8]. These misfolded proteins formed within necrotic cells become exposed as a consequence of plasma membrane disruption and acted as a ligand for tissue-type plasminogen activator (t-PA) and plasminogen [7,8]. Efficient t-PA-mediated plasmin formation leads to the subsequent proteolytic degradation of the dead cell corpse [7,8]. *In vivo* support for this mechanism came from the observation of exaggerated accumulation and impaired removal of misfolded proteins in plasminogen^-/-^ mice following tissue injury [7]. An additional component to the removal of unwanted proteins is the phagocytic arm of the innate immune system. Hence, both extracellular degrading enzymes and phagocytic responses contribute to the removal of dead cells [9,10] and are likely to communicate with each other not only to maximise clearance, but also to minimise self-recognition and maintain tissue homeostasis. Two main cell types mediate the phagocytic clearance of dead cells: macrophages and dendritic cells. Previous studies have shown that plasmin has a pro-inflammatory influence on macrophages [11–15] and increases their capacity to phagocytose apoptotic cells [16]. Dendritic cells, as well as eliminating dead cells, are also a specialised cell type capable of initiating damaging adaptive immune responses to self-antigens. Little is known about the potential of plasmin to alter the unique endocytic potential (including phagocytosis) of dendritic cells.

Here we report that plasmin formed on necrotic cells promotes their phagocytosis by human dendritic cells. This effect was distinct from the ability of plasmin to proteolytically degrade necrotic material, as plasmin also increased the phagocytosis of protease-resistant microparticles. Indicative of a broad-acting mechanism, plasmin also increased the phagocytic function of multiple mouse dendritic cell types *in vitro* and *in vivo*. Plasmin-treated dendritic cells did not undergo maturation and showed reduced migration to the draining lymph node. Furthermore, plasmin-treated dendritic cells had an attenuated capacity to trigger allogeneic lymphocyte expansion. Taken together, our findings support the notion that misfolded proteins formed during necrosis represent a *bona fide* DAMP that activates plasmin and thereby promotes the proteolytic and phagocytic removal of dead cells. Given the pro-inflammatory role of plasmin, we propose that the ability of plasmin to simultaneously suppress the immune response would be relevant during sterile tissue injury where large-scale protective inflammatory responses need to occur alongside the immunologically-discrete removal of cell debris.

## Materials and Methods

### Materials

Reagents were from Life Technologies unless indicated otherwise. Recombinant human t-PA was Actilyse (Boehringer, Ingelheim, Germany). Human plasminogen, human fibrinogen and bovine thrombin were from Merck Millipore (Kilsyth, Victoria, Australia). Human and mouse plasmin were from Hematologic Technologies (Essex Junction, Vermont, USA). Thiazine Red, staurosporine, 6-aminocaproic acid, lipopolysaccharide (LPS), aprotinin, PKH26 and PKH67 fluorophores were from Sigma-Aldrich (St. Louis, Missouri, USA). Recombinant human/mouse IL-4 (rIL-4) and recombinant human/mouse GM-CSF (rGM-CSF) were from Peprotech (Rocky Hill, New Jersey, USA). Ficoll-Paque was from GE Healthcare (Rydalmere, New South Wales, Australia). [H^3^]-thymidine was from Amersham (Little Chalfont, Buckinghamshire, U.K.). Protease inhibitor tablets for cell lysis were from Roche (Mannheim, Germany).

### Animals and human cells

#### Ethics Statement

Experiments were performed on male C57/Black6 mice (6–10 weeks of age). Mice were euthanized with urethane. Animal procedures were conducted in accordance with the Australian National Health and Medical Research Council guidelines and were approved by the institutional Precinct Animal Ethics Committee (PAC). Buffy coats were obtained from blood donations of healthy donors were conducted in accordance with the Declaration of Helsinki, and kindly provided by the Australian Red Cross Blood Service. Their use for this project was approved by Monash University’s Standing Committee on Ethics in Research Involving Humans.

#### Human monocyte-derived dendritic cell cultures (MoDCs)

Peripheral blood mononuclear cells (PBMCs) were isolated from buffy coats using Ficoll-Paque density gradient centrifugation according to manufacturer’s instructions. Heat inactivated autologous serum for cell culture was prepared from platelet-rich plasma (PRP) that was clotted with 10% CaCl_2_ for 3 h at 37°C. After clotting had occurred, the serum was collected and heat-inactivated at 57°C for 30 min. PBMCs (3x10^7^) were cultured in 6 mL of serum-free RPMI media per 25 cm^2^ flask. After 2 h, the cells were gently agitated and non-adherent cells (lymphocytes) were removed. Adherent cells (monocytes) were washed and incubated with 6 mL of AIM-V media plus 1% heat-inactivated autologous serum, 200 U/mL human rIL-4 and 500 U/mL human rGM-CSF for 4–5 days under humidified 5% CO_2_ conditions.

### Microscopy

Phase-contrast micrographs were taken with a Leica DM-IRB microscope. Camera: Hamamatsu ORCA-AG. Objective: NPLAN 40x, 0.55 NA. Acquisition software was MetaMorph v.7.5 (Molecular Devices, Sunnyvale, CA, USA). Images were processed with ImageJ v.1.42q (National Institute of Health). Confocal micrographs were taken on a Nikon A1r-si resonant scanning confocal system (microscope: Nikon Ti; objective: Apo LWD, 40x magnification, 1.15 numerical aperture, water immersion; sequential excitation: 405 nm, 488 nm and 546 nm laser lines; respective emission filters: 450/50 nm, 525/50 nm and 595/50 nm; photomultiplier tube detectors; acquisition software: NIS elements Advanced Research). Images were processed with ImageJ v.1.47q (National Institute of Health).

### Particle uptake assays

MoDCs were resuspended at 1x10^6^ cells/mL in serum-free AIM-V media. Fluorescent polystyrene particles (40 nm and 500 nm; Cat. #F8795 and F8813) were dialysed against phosphate-buffered saline (PBS) and added to MoDCs (1x10^6^cells/mL) in serum-free AIM-V media. A ratio of 512 particles/cell (for MoDCs) and 52 particles/cell (for bone-marrow derived mouse dendritic cells [BM-mDCs]) were used for the 500 nm particles. A ratio of 80,000 particles/cell was used for the 40 nm particles. Particle uptake by MoDCs was analysed by flow cytometry (BD FACS Calibur; BD Biosciences; San Jose, CA, USA).

### Dead cell phagocytosis assay

MoDCs (2x10^6^) were washed in PBS and resuspended in 100 μL of diluent C (supplied with the PKH26 dye). PKH26 (100 μL of 4 μM) was added and incubated for 5 min at room temperature. Heat-inactivated foetal calf serum (FCS; 200 μL) was added and incubated for 1 min. RPMI media (400 μL) containing 10% FCS was then added and centrifuged (1200x*g*, 5 min). Cells were washed twice with RPMI media +10% FCS and then twice with PBS. Cells were resuspended at 1x10^6^ cells/mL in serum-free AIM-V media and plated in 24-well plates (500 μL/well). Next, 2x10^6^ Jurkat T lymphocytes were labelled with PKH67 using the same protocol as above. Cells were then resuspended in PBS at 1x10^6^ cells/mL and incubated at 56°C for 30 min to cause necrosis. Cells were then centrifuged (1600x*g*, 5 min) and resuspended in serum-free RPMI media. Cells were incubated with proteases, LPS and/or aprotinin (see Fig legends) for 15 min at room temperature. Cells were then washed twice with PBS and resuspended at 5x10^6^ cells/mL in serum-free AIM-V media.

PKH67-labelled dead cells (500 μL) were then mixed with PKH26-labelled MoDCs (1:5 ratio of MoDCs to dead cells) for 24 h. Co-culture media was then removed and 500 μL of 0.25% (w/v) trypsin added to dissociate dendritic cells from non-engulfed dead cells. Dissociated MoDCs were centrifuged (1200xg, 5 min), resuspended in 200 μL AIM-V media and double-positive events (i.e. PKH26 and PKH67 positive MoDCs) analysed by flow cytometry (BD FACS Calibur).

### Bone-marrow derived mouse dendritic cells (BM-mDCs)

Bone marrow was harvested from the femur and tibias of mice [17]. Harvested cells were resuspended and incubated in ammonium chloride buffer (155 mM NH_4_Cl, 0.1 mM Na-EDTA, 10 mM KHCO_3_) for 1 min to lyse red blood cells. Cells were centrifuged (1200x*g*, 5 min) and resuspended in RPMI media and passed through a 100 μm strainer (BD Biosciences; San Jose, CA, USA). The resultant cells were cultured at 1x10^6^ cells/mL in complete medium (RPMI media +10% FCS, 4 mM L-glutamine, 100 U/mL streptomycin/penicillin, 20 mM HEPES pH7.4) supplemented with 20 ng/mL mouse rGM-CSF and 20ng/mL mouse rIL-4. Cultures were maintained at 37^°^C under humidified 5% CO_2_ conditions for 6 days before experimentation.

### Immunoblotting

Immunoblotting followed our prior protocol [8]. The primary antibodies used were goat anti-t-PA (Santa Cruz Biotechnology, Dallas USA, sc-5239; 1:1000 dilution), sheep anti-plasminogen (Serotec Kidlington, UK, Code: 7440-0104P 1:1000 dilution), mouse anti-GAPDH (Merck Millipore, MAB374; 1:1000 dilution) and mouse anti-Annexin A2 (BD Biosciences; 1:1000 dilution).

### Plasmin inactivation

Plasmin was inactivated by incubation with a 1000-fold molar excess of D-Val-Phe-Lys-chloromethyl ketone (Calbiochem Merck Millipore, Kilsyth, Victoria, Australia) at 37°C for 30 min then dialyzed overnight at 4°C in PBS, 0.2 μm-filtered and its concentration determined (relative to the original active plasmin batch) by densitometry of Coomassie-stained sodium dodecyl sulphate-polyacrylamide gel electrophoresis (SDS-PAGE) whereby titration of inactive plasmin was compared to titration of a known concentration of plasmin via SDS-PAGE electrophoresis. Loss of proteolytic activity was verified by amidolytic assay [18].

### Cell Viability assays

The membrane integrity of MoDCs was assessed as previously described [19] using the CytoTox-96 NonRadioactive Cytotoxicity Assay (Promega, Madison, WI, USA) and the viability using the CellTiter-96 AQueous Non-Radioactive Cell Proliferation Assay (Promega, Madison, WI, USA) according to the manufacturer's instructions.

### Kinomic array and computational pathway analysis

MoDCs were incubated in the presence/absence of 100 nM plasmin. After 3 hours of incubation, cell homogenates were prepared and sent to Kinexus Bioinformatics (Vancouver, BC, Canada) for blinded kinomic analysis using the KAM-1.2 chip equipped with ~500 pan-specific and ~300 phospho-site specific antibodies as previously described [19]. The short list of differentially regulated events identified by the KAM-1.2 chip was subjected to two independent forms of computational pathway analyses: the first was via Ingenuity Pathway Analysis where the analyst (from the Australian Proteome Analysis Facility) was blinded to both the experimental design and the overall project hypothesis, the second was a batch enquiry of the US National Cancer Institute/Nature Publishing Group-curated Pathway Interaction Database [20].

### Mixed lymphocyte reaction

MoDCs were seeded at 1x10^6^ cells/mL in serum-free AIM-V media and treated with or without plasmin for 24 h. MoDCs were then washed in PBS, counted and seeded at 15,000 cells/well of a round-bottomed 96-well plate. Allogeneic PBMCs (150,000) were then added to each well for 3 days. [H^3^]-thymidine (1 μCi/well) was added and co-cultures were incubated for a further 24 h before [H^3^]-thymidine incorporation was measured using a scintillation counter (Top Count; Packard Instrument, Meriden, CT). In-house control experiments verified that the responder cells were viable and responsive to stimulation in these experiments.

### Triton X100-insoluble protein extraction

Triton-insoluble proteins were extracted as previously described [7]. Note that in these experiments, the same number of cells was seeded into the tissue culture plates. After treatment, protein lysates were obtained using a constant volume of lysis buffer.

### Cytokine quantification

Conditioned media of MoDCs was assessed using the Cytometric Bead Arrays, specifically the ‘Human Th1/Th2/Th17 Cytokine Kit’, the ‘Human TGF-β1 Single Plex Flex Set Kit’ and the ‘BD OptEIA Human IL-10 and IL-12 (p40) ELISA kits II’ according to the manufacturer’s instructions (BD Biosciences; San Jose, CA, USA).

### Intradermal microparticle injection

The base of an adult mouse tail was injected with 100 μL of the stipulated reagents (dialysed against sterile 0.35 M HEPES pH7.4) with or without microparticles (1% w/v) using a 25-gauge needle. Inguinal lymph nodes were harvested 24 h later for cell staining and flow cytometry (BD LSR II analyser).

### Flow cytometry

Unless otherwise stated, all antibodies used for flow cytometry were from BD Biosciences (San Jose, CA, USA). For assessment of the maturation of human MoDCs, the antibodies used were: PE/APC-conjugated mouse anti-CD40 (clone 5C3), PE-conjugated mouse anti-CD70 (clone Ki-24), PE/Biotin-conjugated mouse anti-L307.4, FITC/APC-conjugated mouse anti-HLA-DR (clone G46-6[L243]), FITC-conjugated mouse anti-Human CD274 (clone MIH1), PE/APC/Biotin-conjugated mouse IgG1 isotype control (clone MOPC-21) and PE/APC/Biotin-conjugated mouse IgG2a isotype control (clone G155-178). For the phenotyping of cells in BM-mDCs and in mouse draining lymph nodes, the antibodies used were: PE-conjugated rat anti-CD4 (clone GK1.5), PerCp-conjugated rat anti-CD8α (clone 53–6.7), PerCp/PECy7-conjugated rat anti-CD11b (M1/70), V450-conjugated Armenian Hamster anti-CD11c (clone HL3), PE-conjugated rat anti-CD86 (clone GL1), PE-conjugated rat anti-CD103 (clone M290), APC-conjugated rat anti-CD172a (clone P84), Biotin/IC-conjugated rat anti-CD207 (from Dendritics, Lyon, France; clone 929F3.01) PerCP/Cy5.5-conjugated rat anti-Gr-1 (clone RB6-8C5) and APC/Cy7-conjugated rat anti-MHC Class II (clone M5/114.15.2). Flow cytometry data was analysed using FlowJo software version 9.4 (Tree Star Inc, Ashland, OR, USA).

### Statistical Analyses

Statistical analyses (1-way ANOVA, two-tailed Student’s t-test or Grubbs’ test; see Fig legends) were performed with GraphPad Prism v.6.01 (S5 Fig). A p-value <0.05 was considered as statistically significant.

## Results

### Plasmin increases the phagocytic capacity of human MoDCs

**Fig 1:**
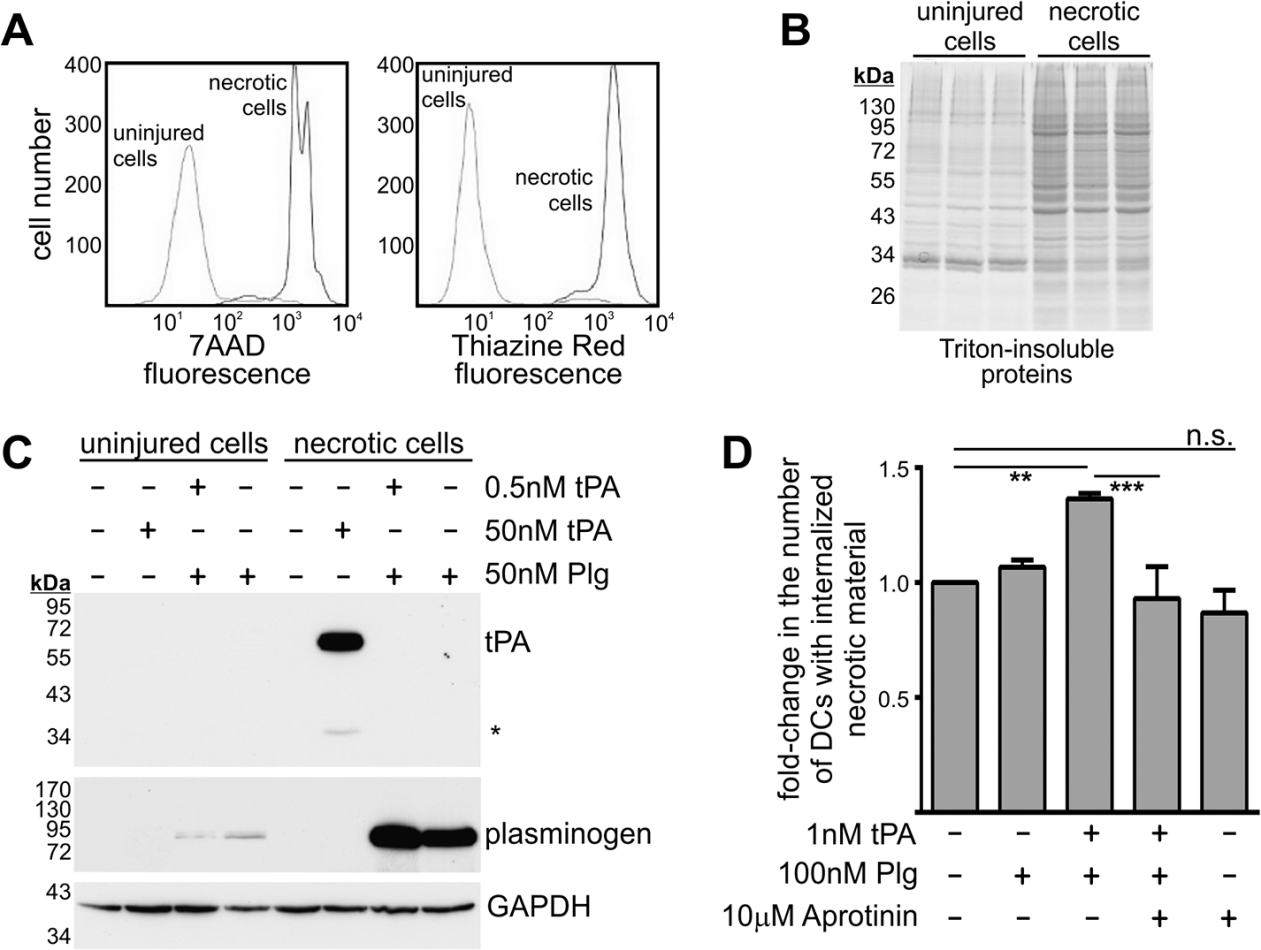
Necrotic cells harbour misfolded proteins and generate surface-bound plasmin which increases their phagocytosis by human MoDCs. (A) Uninjured and necrotic Jurkat lymphocytes were stained with 10 mg/L 7AAD and 10 mg /L Thiazine Red for 15 min then subjected to flow cytometry. (B) The Triton-insoluble fractions of uninjured and necrotic Jurkat lymphocytes were subjected to SDS-PAGE under reducing conditions and subsequent Coomassie staining. (C) Uninjured and necrotic Jurkat lymphocytes were incubated with t-PA and/or plasminogen for 15 min and washed. Total cellular protein lysates were prepared and subjected to SDS-PAGE under reducing conditions and subsequent immunoblot analysis. Most of the t-PA used was in single-chain form (~70 kDa), however a small extent of two-chain t-PA was also apparent (the band indicated by the asterisk at ~35 kDa). (D) PKH67-labelled necrotic Jurkat lymphocytes were treated with the indicated reagents for 15 min, then washed and incubated with PKH26-labelled human MoDCs. 24 h later, the proportion of double-positive (PKH67^positive^ and PKH26^positive^) MoDCs was assessed by flow cytometry. Data are displayed as fold-change in double-positive MoDCs (mean ± s.e.m.; n = 3–5 independent experiments). Data was normalized to the group where necrotic cells received no exogenous reagent. **p<0.01 and ***p<0.001 by 1-way ANOVA with Newman-Keuls post-hoc analysis.

We have previously shown that misfolded proteins formed by dead cells are a cofactor and substrate for t-PA-mediated plasmin formation [7,8]. We now wanted to determine whether plasmin formed on the surface of dead cells also influences their clearance via phagocytosis. Before addressing this question, we first characterised necrotic target cells for *in vitro* phagocytosis and their capacity to bind t-PA and plasminogen (Fig 1A–1C). Necrotic cells were generated by heat-treating human Jurkat lymphocytes. Necrosis was confirmed by the binding of 7AAD (a membrane-impermeable dye; Fig 1A) while the presence of misfolded proteins was demonstrated by the binding of Thiazine Red (a dye selective for misfolded proteins; Fig 1A) and by an increase in the abundance of detergent-insoluble proteins (Fig 1B). Lastly necrotic Jurkat cells bound substantially more exogenous t-PA and plasminogen than uninjured Jurkat lymphocytes (Fig 1C). PKH67-labelled Jurkat cells were heat-treated to induce necrosis, then incubated with t-PA and plasminogen (alone or in combination), washed to remove unbound material, and then incubated with PKH26-labelled MoDCs. The extent of phagocytosis was determined by flow cytometry 24 h later. Co-treatment of necrotic cells with t-PA and plasminogen increased their phagocytosis (Fig 1D). Importantly, no increase in phagocytosis was observed when necrotic cells were co-treated in the presence of aprotinin, a plasmin inhibitor (that only weakly inhibits t-PA). Thus, the proteolytic activity of plasmin promotes the phagocytosis of necrotic cells. Hence, t-PA-mediated plasmin formation on the surface of necrotic cells promotes their phagocytosis by MoDCs.

### Plasmin increases the capacity of MoDCs to phagocytose microparticles

**Fig 2:**
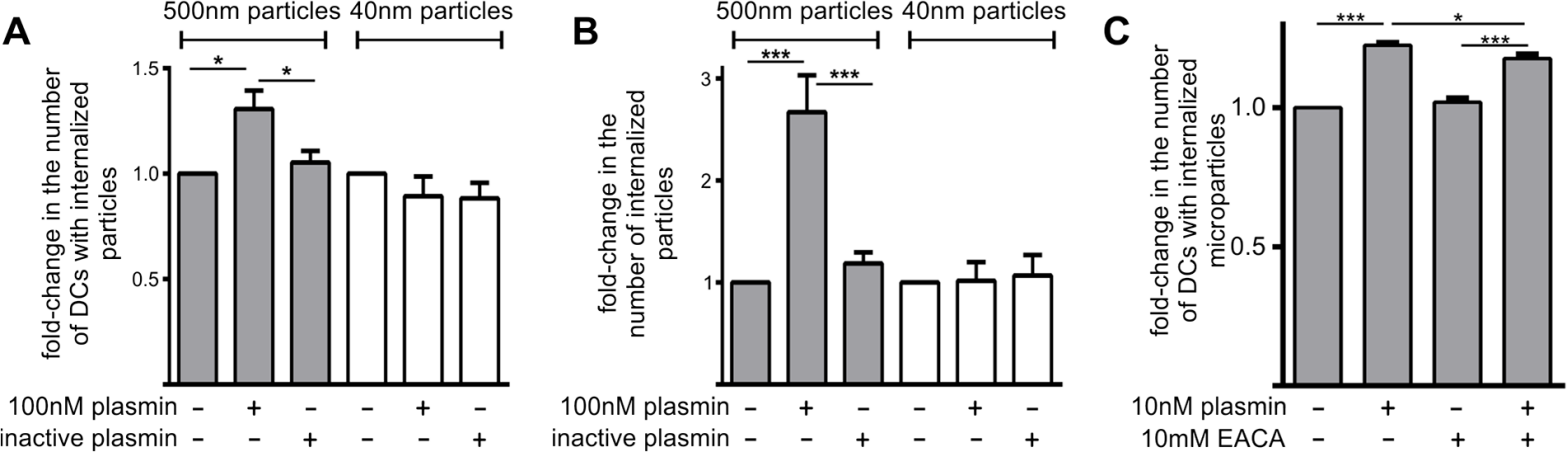
The proteolytic activity of plasmin increases the phagocytosis of microparticles, but not nanoparticles, by MoDCs. MoDCs were treated with 500 nm fluorescent microparticles or 40 nm fluorescent nanoparticles in the presence/absence of 100 nM active or inactive plasmin. 3 h later the relative number of particle-positive MoDCs (Panel A) and internalized particles (Panel B) were assessed by flow cytometry. Data shown are mean ± standard error (n = 3–4 independent experiments). Data were normalized to the groups where MoDCs received neither plasmin nor inactive plasmin. *p<0.05 by 1-way ANOVA with Newman-Keuls post-hoc analysis. (C) MoDCs were treated with 500 nm fluorescent microparticles in the presence/absence of 10 nM active plasmin and/or 10 mM EACA. The relative number of particle-positive MoDCs was assessed by flow cytometry 24 h later. Data are displayed as mean fold-change in particle positive MoDCs ± s.e.m. (n = 4 independent experiments). Data was normalized to MoDCs receiving neither plasmin nor EACA. *p<0.05, **p<0.01 and ***p<0.001 by 1-way ANOVA with Newman-Keuls post-hoc analysis.

The observed increase in phagocytosis could be due to a plasmin-mediated degradation of misfolded proteins within necrotic cells [7,8], or to a direct interaction of plasmin with MoDCs. To distinguish between these two possibilities, we evaluated the effect of plasmin (100 nM) on the phagocytosis of protease-resistant fluorescent microparticles (500 nm diameter). After 3 h of co-incubation, plasmin significantly increased both the number of microparticles per cell (Fig 2A) and the number of MoDCs with internalized microparticles (Fig 2B). Hence, this pro-phagocytic function of plasmin does not rely upon proteolytic degradation of the phagocytic target. Importantly, catalytically inactive plasmin had no effect on microparticle uptake after 3 h (Fig 2A and 2B). Treatment of MoDCs with a 10-fold lower concentration of plasmin (10 nM) similarly increased microparticle uptake after 3 h (not shown) and 24 h (Fig 2C) of co-incubation. Interestingly, the observed plasmin-mediated increase in microparticle uptake was only mildly attenuated by EACA (a lysine analogue) after 24 h (Fig 2C) of co-incubation. This finding suggests that plasmin may not solely rely on kringle-mediated interactions with dendritic cell-surface receptors to enhance phagocytic function.

### Plasmin does not increase the uptake of nanoparticles

Previous studies have shown that the uptake of microparticles by dendritic cells is an actin-dependent process, whereas the uptake of nanoparticles (40 nm in diameter) uses cholesterol/caveolae and clathrin-mediated endocytic mechanisms [17,21–23]. Accordingly, we assessed whether plasmin increased the endocytic uptake of nanoparticles. 3 h treatment of MoDCs with plasmin failed to increase nanoparticle uptake (Fig 2A and 2B). Hence, the ability of plasmin to promote particle uptake is selective for the actin-dependent pathway of phagocytosis, and not the result of a general enhancement of endocytic capacity.

### Plasmin increases TGF-β expression by MoDCs without affecting maturation or viability

**Fig 3:**
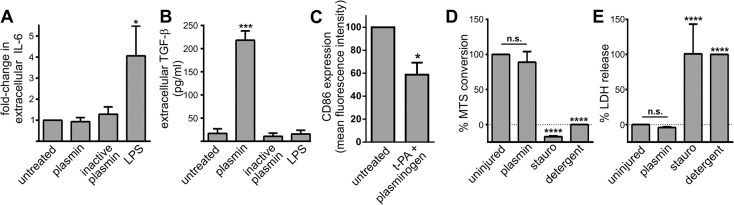
Plasmin-treatment increases TGF-β release, but not the maturity or viability of MoDCs. A and B) MoDCs were treated with 10 nM active plasmin or inactive plasmin or 500 ng/mL LPS. After 48 h, the concentration of IL-6 (Panel A) and TGF-β (Panel B) in the conditioned media was determined. Data are displayed as mean ± s.e.m (n = 4 independent experiments). IL-6 data was normalized to untreated MoDCs. *p<0.05 and ***p<0.001 by 1-way ANOVA with Newman-Keuls post-hoc analysis. C) MoDCs were incubated in the presence/absence of 1 nM t-PA+100 nM plasminogen. 24 h later the cell surface expression of CD86 was assessed via flow cytometry. The mean fluorescence intensity of the CD86 signal was normalized to that of untreated MoDCs. Data are displayed as mean ± s.e.m (n = 3 independent experiments). *p<0.05 by 2-tailed Welch’s unequal variances t-test. D and E) MoDCs were treated with 100 nM plasmin or 200 nM staurosporine (stauro) for 24 h. Cellular metabolism (Panel C) and plasma membrane integrity (Panel D) were assessed by the MTS and LDH assays respectively. Data was normalized to the values for untreated cultures and detergent-treated cultures. Data are shown as mean ± s.e.m. (n = 2 for staurosporine-treatment and n = 5 independent experiments for all other groups). ****p<0.0001 by 1-way ANOVA with Newman-Keuls post-hoc analysis.

We next determined whether plasmin was having a broader influence on dendritic cells by assessing the maturation, cytokine levels, morphology and viability of MoDCs. To assess MoDC maturation status, we measured the extracellular levels of an array of cytokines. These studies showed that plasmin-treatment alone did not alter IL-6 (Fig 3A), IL-10 or IL-12 levels (S1 Fig) suggesting that plasmin does not cause MoDC maturation. In contrast, the maturation of MoDCs via LPS-treatment coincided with significant increases in IL-6 (Fig 3A), IL-10 and IL-12 (S1 Fig). Plasmin-treatment did however trigger a ~13-fold elevation in extracellular TGF-β levels (Fig 3B); which was seemingly unrelated to the maturation status of MoDCs given that LPS-treatment failed to alter TGF-β levels (Fig 3B). To further examine whether plasmin affects MoDC maturation, we monitored the expression of several immunomodulatory cell-surface markers. Consistent with the notion that plasmin does not cause dendritic cell maturation, plasmin generation was found to decrease expression of CD86, CD70, CD80, CD274 and HLA-DR in MoDCs (Fig 3C and S1 and S4 Figs. The observed plasmin-mediated suppression of multiple distinct markers suggests that plasmin proteolyses (or downregulates) an array of cell-surface immunomodulatory receptors, consistent with its ability to maintain an immature phenotype.

We also noted that plasmin elicited a pronounced change in dendritic cell morphology (S2 Fig). Further characterisation showed that this change in cell morphology was rapid (occurred within 3 h), sensitive (caused by 0.1 nM plasmin), dependent upon proteolytic activity (inhibited by aprotinin), and only partially mediated by lysine-binding (mildly attenuated by EACA) (n = 3; not shown). The observed morphological change did not lead to MoDC-detachment (n = 3; not shown); unlike other forms of plasmin-mediated cellular rearrangement [24,25] and may instead be related to the migratory influence of plasmin on dendritic cells [26]. Importantly, the observed plasmin-mediated alterations in MoDC morphology/function were not the result of cellular toxicity, as no reduction in metabolism (Fig 3D) or perturbation of the plasma membrane (Fig 3E) was detected. Collectively, plasmin produces a discrete influence on dendritic cells: by selectively promoting phagocytosis, increasing TGF-β expression, decreasing immunomodulatory receptor expression and altering cell morphology, without causing overt stress or maturation of MoDCs.

### Plasmin-treated MoDCs fail to efficiently stimulate an allogeneic response

**Fig 4:**
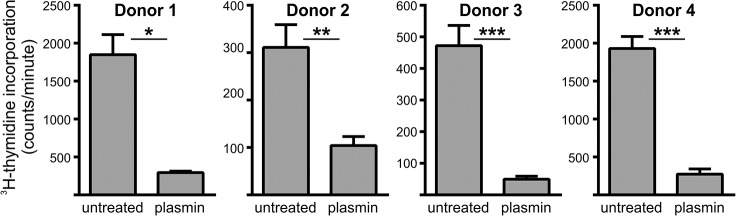
Plasmin-treatment of MoDCs suppresses allogeneic lymphocyte proliferation. MoDCs prepared from four different donors were treated with 100 nM plasmin for 24 h, then washed and incubated with allogeneic PBMCs in triplicate for 4 days. 3H-thymidine incorporation over the last 24 h was taken as a measure of lymphocyte proliferation. Data are shown as mean ± s.e.m. *p<0.05, **p<0.01 and ***p<0.001 by unpaired two-tail Student’s t-test.

The observation that plasmin-treatment markedly increased total TGF-β levels was intriguing since plasmin is known to proteolytically activate TGF-β [27], and because TGF-β is a potent immunosuppressant of downstream lymphocyte activation [28,29]. We therefore determined whether plasmin impaired the capacity of MoDCs to mount an adaptive immune response. To this end, mixed lymphocyte reactions were performed using MoDCs and allogeneic leukocytes from 4 independent donors. As shown in Fig 4, plasmin-treated MoDCs had significantly reduced capacity to stimulate a mixed lymphocyte reaction. Hence, plasmin dramatically attenuates the ability of MoDCs to promote an allogeneic immune response.

### Identification of putative immunomodulatory signals induced by plasmin

**Fig 5:**
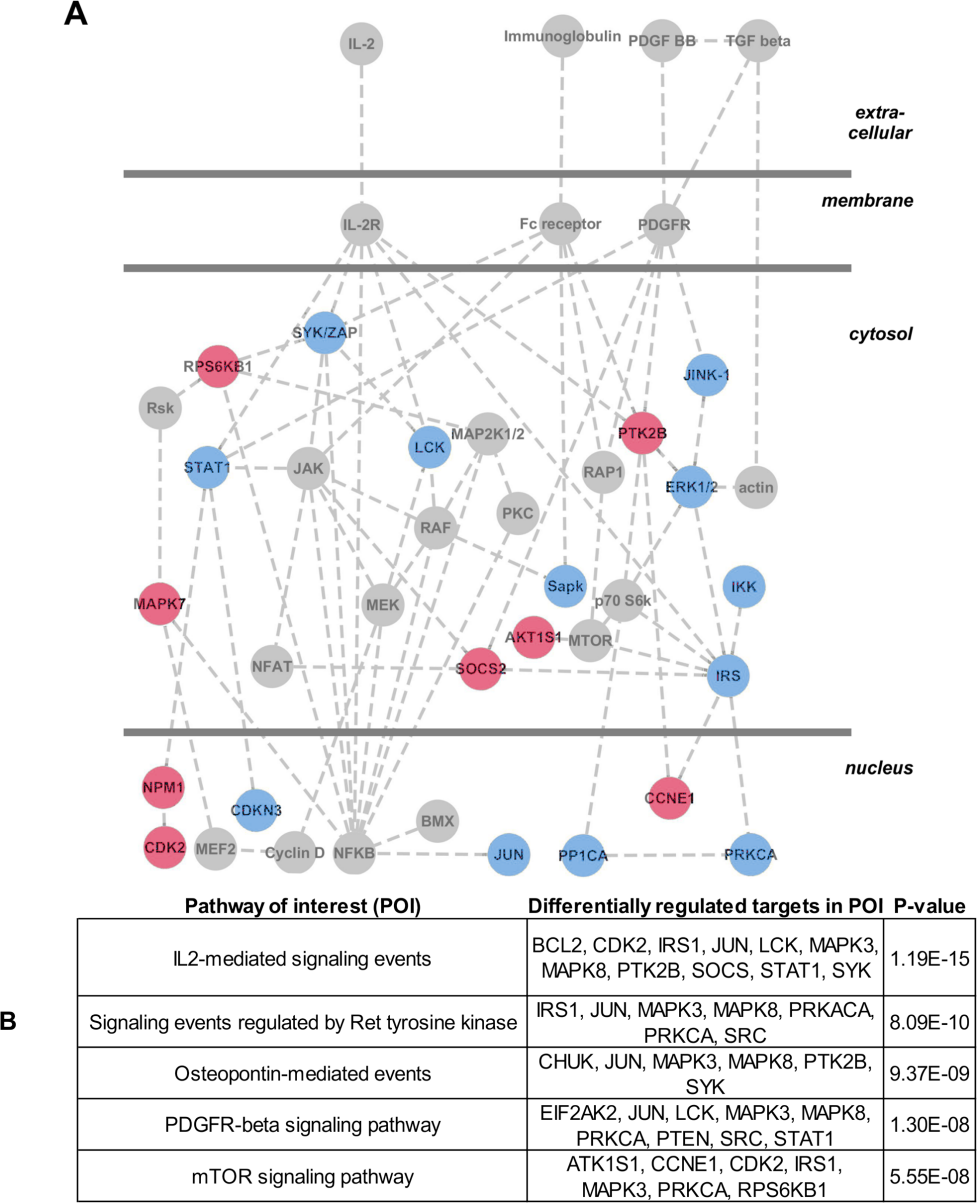
Kinomic screening and prediction of the signalling events that underlie plasmin-mediated immunomodulation. (A) MoDCs were incubated in the presence/absence of 1 nM t-PA and 100 nM plasminogen. 3 h later cultures were lysed and subject to kinomic microarray analysis yielding a short list of 31 signalling proteins that were differentially regulated upon plasmin-treatment (see S1 Table for complete dataset). The short list was subjected to Ingenuity Pathway Analysis to yield three canonical signalling pathways (not shown) for plasmin-mediated immunomodulation, which were manually merged and refined to generate a single putative signalling pathway that underlies plasmin-mediated immunomodulation. Red symbols represent upregulated events. Blue symbols represent downregulated events. (B) The kinomic short list of differentially regulated signalling proteins was batch analysed using the Pathway Interaction Database (PID). Shown are the 5 most significantly perturbed pathways of interest (POI), the differentially regulated proteins within each POI and the degree of statistical significance (p-value computed using the default PID hypergeometric distribution test).

Prior studies show that plasmin can modulate dendritic cell function by cleaving cell-surface Annexin A2, which in turn triggers downstream phospho-ERK1/2 (22). Surprisingly, we were unable to detect cleaved Annexin A2 (S1 Fig), or increased phospho-ERK1/2 (not shown) in MoDCs following plasmin-treatment. Accordingly, to assist the future identification of putative signalling events that may underlie plasmin-mediated immunomodulation, we performed a kinomic screen whereby MoDCs were treated with/without plasmin for 3 h, after which cell lysates were harvested and subjected to Kinexus antibody microarray (which utilises ~500 pan- and ~340 phospho-specific antibodies). Pair-wise comparison of the microarray data produced a high-confidence list of 31 signalling proteins that were differentially regulated in MoDCs by plasmin (S1 Table). This kinomic dataset was then subjected to Ingenuity Pathway Analysis where the analyst was blinded to both the experimental design and the overall project hypothesis. The top three canonical pathways identified by the Ingenuity Pathway Analysis were collated into a single hypothetical signalling network underlying plasmin-mediated immunomodulation (Fig 5A). These analyses suggest that plasmin-mediated immunomodulation involves altered platelet-derived growth factor (PDGF) receptor signalling, IL-2 receptor signalling and Fc-receptor signalling. Independent analysis of the kinomic data via the Pathway Interaction Database further supports the suggestion that plasmin alters signalling downstream of the PDGF and IL-2 receptors (Fig 5B). Altogether, our kinomic data suggests that future studies should assess whether PDGF and IL-2 signalling in dendritic cells can be modulated by plasmin activity; especially within the context of phagocytosis and immunomodulation.

### Plasmin increases the uptake of microparticles by mouse dendritic cells

We next determined whether plasmin could also promote the phagocytic function of mouse dendritic cells. To this end, bone marrow-derived mouse dendritic cells (BM-mDCs) were incubated with fluorescent microparticles in the presence or absence of mouse plasmin. After 6 h of co-incubation, the extent of microparticle uptake was assessed within conventional CD11c^+^ dendritic cells. As shown in S3 Fig, plasmin caused a significant increase in the number of CD11c^+^ dendritic cells with internalized microparticles. Altogether, our results show that plasmin-treatment increases the phagocytic capacity of both human and mouse dendritic cells *in vitro*.

### Plasmin formation increases microparticle uptake by dendritic cells *in vivo*

**Fig 6:**
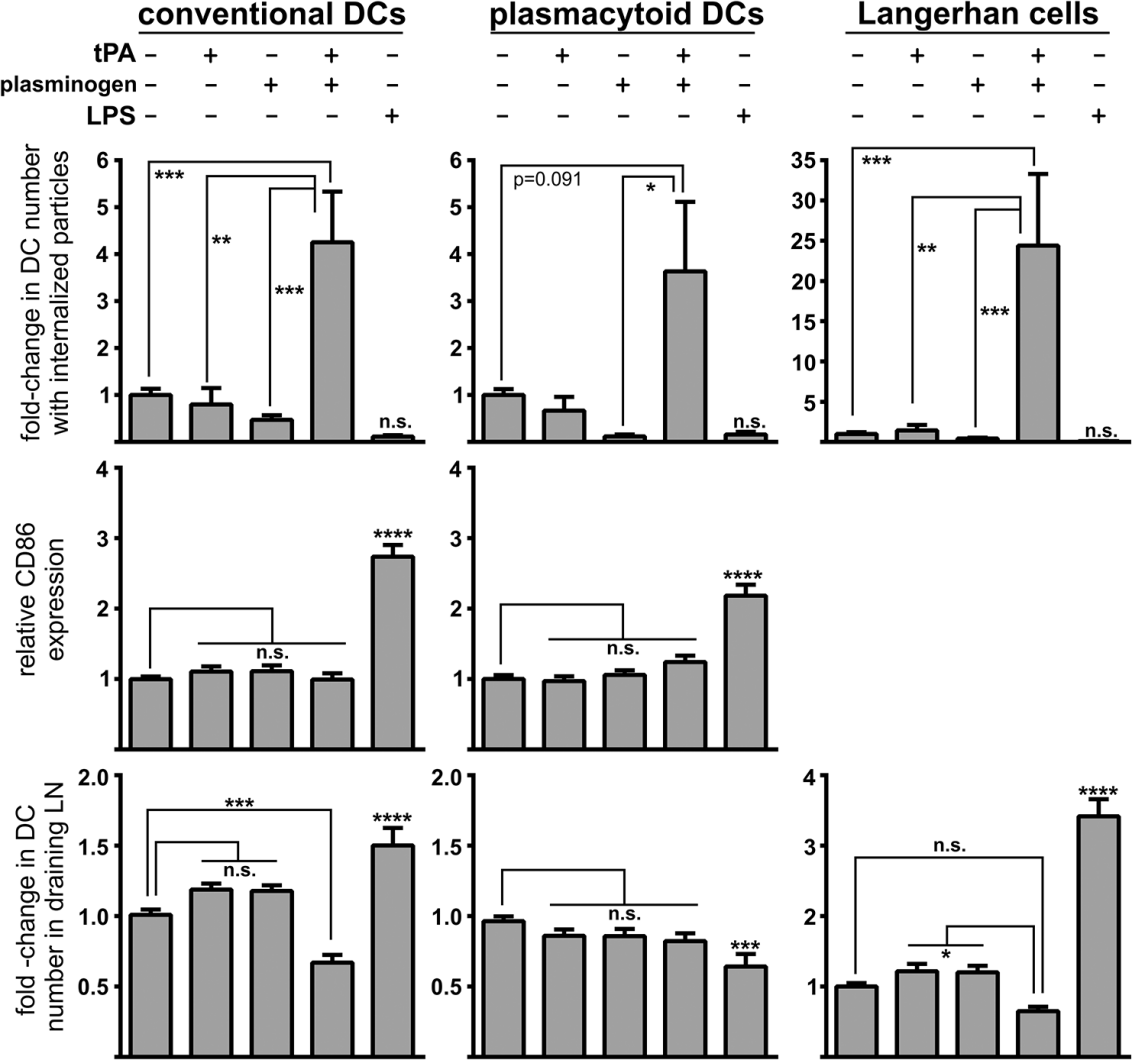
Intradermal plasmin injection increases the phagocytosis of microparticles by multiple mouse dendritic cell types *in vivo*, but does not trigger their maturation or migration to the draining lymph nodes. Mice were intradermally injected with microparticles in the presence/absence of 0.02 pmol of t-PA, 0.1 pmol of plasminogen or 10 μg of LPS. 24 h after injection, the draining inguinal lymph nodes were harvested and single cell suspensions were stained for cell-surface markers (CD11c, CD11b, MHC class II, CD86 and B220) and subjected to flow cytometry. Events were gated into three populations: CD11c^positive^ MHC class II^positive^ conventional dendritic cells (*left column*), CD11b^positive^ B220^positive^ plasmacytoid dendritic cells (*middle column*) and CD11c^positive^ MHC class II^positive^ CD11b^positive^ CD207^positive^ CD103^negative^ Langerhans cells (*right column*, note that CD86 analysis was not included for Langerhans cells). Within each dendritic cell population the percentage of cells with internalized particles (*top row*), their maturation status as determined by relative mean fluorescence intensity of CD86-staining (*middle row*), and their number relative to all cells within the draining lymph nodes (LN; *bottom row*) were determined. Data are shown as mean ± s.e.m. (n = 8–11 independent experiments). *p<0.05, **p<0.01, **p<0.001 and ***p<0.0001 by 1-way ANOVA with Newman-Keuls post-hoc analysis. Two outliers were identified by Grubb’s test and excluded from the analysis.

To explore whether plasmin could similarly modulate dendritic cell function *in vivo*, fluorescent microparticles were injected intradermally into the base of the tail of wild-type mice in the presence or absence of t-PA and plasminogen (alone or in combination). In addition, mice were co-injected with LPS and microparticles to serve as a positive control for enhanced maturation and migration by dendritic cells. Cells within the draining lymph nodes were collected 24 h after injection, and three major dendritic cell types (conventional, plasmacytoid and Langerhans cells) were stained and assessed for microparticle uptake, maturation status and overall number. Consistent with our *in vitro* data, plasmin generation increased the *in vivo* phagocytic capacity of all three dendritic cell populations (Fig 6; top panels). Moreover, this increase in phagocytosis did not result in maturation, as determined by CD86 expression (Fig 6; middle panels). Plasmin generation also caused a significant reduction in the number of conventional dendritic cells (cDCs) entering the draining lymph nodes with similar albeit non-significant trends seen for plasmacytoid dendritic cells (pDCs) and Langerhans cells (Fig 6; bottom panels). The reason for a plasmin-mediated reduction in dendritic cell migration to the draining lymph nodes may relate to plasmin acting as a chemotaxic agent [26,30], whereby plasmin discourages the migration of dendritic cells away from the injection site. Injection of either t-PA or plasminogen alone failed to alter any of the measured dendritic cell parameters indicating that the observed effects require plasmin formation. As expected, and in contrast to the influence of plasmin, LPS injection caused maturation and reduced microparticle uptake in all dendritic cell subtypes. LPS also increased migration of conventional dendritic cells and Langerhans cells to the draining lymph nodes (Fig 6). These *in vivo* observations are consistent with the notion that plasmin modulates dendritic cells by increasing their phagocytic capacity in a manner that simultaneously avoids their maturation and migration to draining lymph nodes.

## Discussion

**Fig 7:**
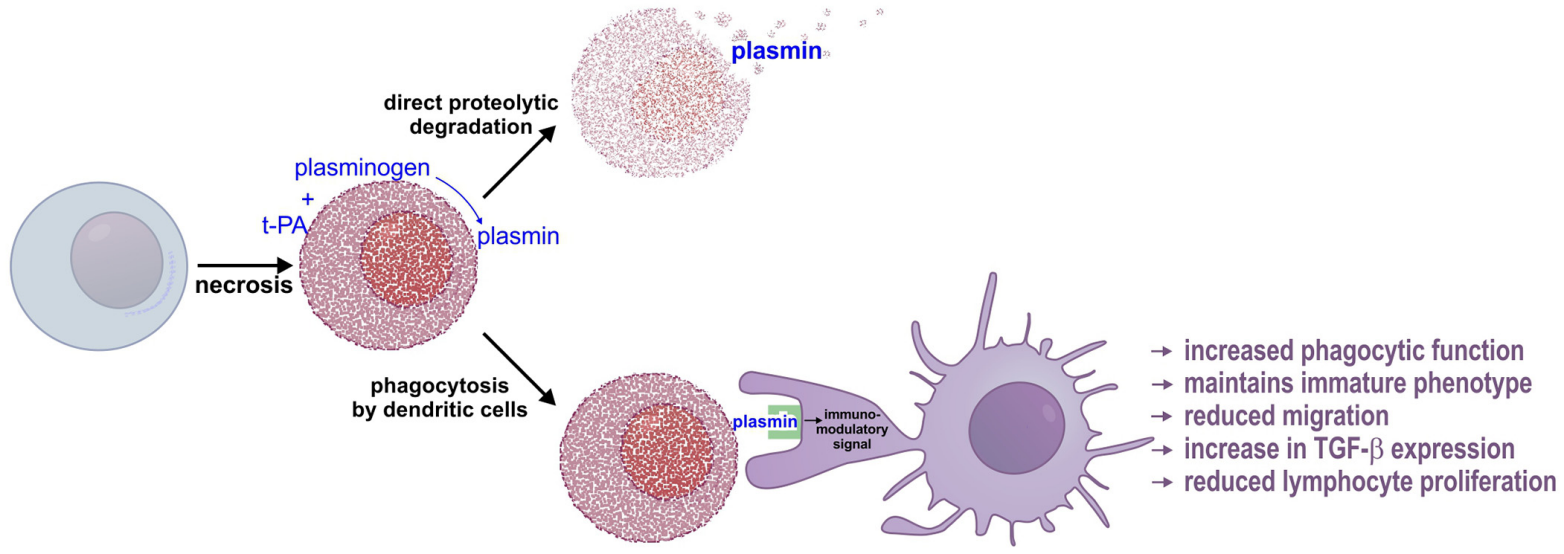
Working model for plasmin-mediated clearance of dead cells. Injury to a live cell results in necrosis and widespread aggregation of intracellular proteins via the phenomenon of *Nucleocytoplasmic Coagulation* (NCC) [7] (in red). Plasma membrane breakdown allows NCC-protein aggregates to bind t-PA and plasminogen and facilitate *in situ* plasmin formation [8]. Plasmin then proteolytically degrades NCC-aggregated proteins. In addition, an immature dendritic cell comes into physical contact with the necrotic cell. Plasmin directly activates dendritic cells which transduce an immunomodulatory signal to dendritic cells that instructs immunologically discrete clearance by increasing phagocytic capacity, increasing TGF-β expression, maintaining an immature phenotype, and preventing migration to draining lymph nodes. The plasmin-stimulated dendritic cell, because of its immature status and because of high TGF-β levels is unable to effectively trigger lymphocyte proliferation and adaptive immunity.

We have previously shown that t-PA-mediated plasmin generation participates in the degradation of dead cells from injured tissues [7,8]. As the removal of dead cells is classically linked to innate immune response [2], we considered whether plasmin also enhances the clearance of dead cells by modulating the phagocytic function of dendritic cells. Our investigation establishes the proof-of-concept that plasmin not only promotes the phagocytic capacity of dendritic cells, but it does so in a manner that avoids dendritic cell maturation and T cell stimulatory activity. Our observations complement the findings that plasmin modulates macrophage function by increasing efferocytosis [16,31] and uptake of aggregated low-density lipoprotein [32]. Fig 7 outlines our working model for how plasmin influences both the phagocytic and non-phagocytic arms of dead cell removal.

Necrosis causes misfolding and aggregation of intracellular proteins via a process called Nucleocytoplasmic Coagulation (NCC) [7]. Breakdown of the plasma membrane during necrosis exposes NCC-protein aggregates, which bind t-PA and plasminogen and promotes *in situ* plasmin formation [8] allowing proteolytic degradation of NCC-aggregated proteins. Independent of direct NCC-proteolysis, an immature dendritic cell comes into physical contact with the plasmin-bearing necrotic cell. Plasmin cleaves and thereby reduces the expression of numerous cell-surface immunomodulatory receptors, which in turn may help maintain an immature phenotype. In addition, we propose the plasmin-mediated cleavage of an unidentified substrate triggers an immunomodulatory signal(s) that increases the phagocytic capacity of dendritic cells (see below).

A surprising observation was that plasmin also attenuated the ability of dendritic cells to mount an adaptive immune response. This was concluded from *in vitro* mixed lymphocyte reactions (Fig 4) and *in vivo* studies as plasmin-induced microparticle uptake after intradermal injection did not result in dendritic cell maturation or increased migration to the draining lymph nodes (Fig 6). Further support for this immunosuppressive effect of plasmin stems from the observation that plasmin-treated dendritic cells dramatically increased their expression/release of TGF-β – a potent immunosuppressant [28,29], but had no effect on the release of IL-10 or IL-12. Hence, we postulate that *in situ* plasmin formation tags phagocytic targets and facilitates their tolerogenic clearance via both non-phagocytic and phagocytic pathways. It would also be interesting to determine whether the capacity of LPS to promote MoDC maturation and/or release of IL-10 or IL-12 could be modulated by plasmin formation. Similarly, it remains to be determined whether IL-10 or IL-12 is produced from DC’s treated with necrotic cells in the presence/absence of t-PA and plasminogen.

Previous studies have shown that plasmin, by acting upon macrophages and neutrophils, promotes inflammation [12–15]. For example, plasmin increases expression of pro-inflammatory IL-6 by macrophages [11]. In contrast, we found that plasmin elicited no change in the release of IL-6 from dendritic cells (Fig 3A). Hence, the actions of plasmin are critically cell type-dependent. The ability of plasmin to promote inflammation yet suppress immune responses has physiological appeal when one considers the scenario of sterile tissue injury—where large-scale protective inflammatory responses need to occur in parallel with an efficient means to remove cell debris without inadvertently triggering autoimmunity.

One salient point is whether endogenous plasmin(ogen) levels increase in the interstitial space to a concentration that influences dendritic cells *in vivo*. Whilst no study has accurately measured interstitial levels of plasmin(ogen), the plasma concentration of plasminogen is high ~2 μM [33] and endogenous levels of plasmin(ogen) in serum are sufficient to increase efferocytosis by macrophages [16]. Hence, it seems likely that the low levels of plasmin(ogen) (0.1–10 nM) used in our *in vitro* experiments are physiological plausible. It should also be noted that plasminogen concentrations can increase ~6-fold within sites of tissue injury [15]. Such injury-induced increases in plasminogen persist for days and correlate with inflammation and healing [15]. Indeed, plasminogen is actively transported to sites of injury by macrophages/neutrophils (rather than by non-specific accumulation due to vessel leakage) [15]. Thus, the selective recruitment of plasminogen by macrophages/neutrophils provides a putative mechanism as to how plasminogen levels can increase to modulate dendritic cell function in the extravascular space *in vivo*.

Our findings and those of others [16,34] suggest that plasminogen^-/-^ mice would be more susceptible to injury-induced autoimmunity. To our knowledge, no study to date has addressed this question. It is interesting to note, however, that increasing plasmin formation (via a lowering of the t-PA-inhibitor protein ‘plasminogen activator inhibitor-1’) suppresses myosin-induced autoimmune myocarditis in rats [35]. Curiously, myosin is both a NCC-prone protein [36] and a cofactor/substrate for t-PA-mediated plasminogen activation [37]. Future studies should now address whether plasmin formation at sites of injury attenuates autoimmunity.

The plasmin-initiated phagocytic signalling pathway was not identified in this study. As plasmin has been shown to trigger phospho-ERK1/2 activation in MoDCs via cleavage of Annexin A2 [26], we assessed whether plasmin-mediated Annexin A2 cleavage occurred under our experimental conditions. Surprisingly, when plasmin was added to intact MoDCs, cleavage of Annexin A2 was only observed when protein lysates were prepared in the absence of protease inhibitors (S2 Fig). Thus, analogous to plasmin-mediated cleavage of the carboxy-terminus of NR1 [38], plasmin-mediated cleavage of Annexin A2 appears to be a non-physiological event that occurs following cell lysis. Moreover, addition of the Annexin A2-derived cleavage fragment failed to recapitulate the influence of plasmin on MoDCs [39] and we were unable to demonstrate increased phospho-ERK1/2 or phospho-Akt following plasmin-treatment of MoDCs (not shown). Hence, Annexin A2 cleavage is unlikely to transduce the effects of plasmin on dendritic cells. Instead, our kinomic analyses suggest that plasmin alters signalling downstream of the Fc, PDGF and IL-2 receptors. While additional studies are required to validate the role of these pathways in promoting phagocytosis or tolerogenicity, some speculation can be considered. It is well known that the Fc receptor performs actin-dependent phagocytosis of targets that have been opsonised with immunoglobulin. However, as our phagocytic experiments (Figs 1, 2 and 6) did not involve opsonisation with immunoglobulin, altered Fc receptor signalling is an unlikely explanation for the pro-clearance effect of plasmin. Similarly, a direct phagocytic role for the widely-studied IL-2 receptor on the dendritic cell-surface has not been reported and thus altered IL-2 receptor signalling also represents an unlikely basis for plasmin-mediated immunomodulation. Interestingly, PDGF receptor signalling also affects the actin cytoskeleton [40] and has been found to directly enhance phagocytic function, albeit in non-dendritic cell types [41,42]. All forms of PDGF and the PDGF-Rβreceptor have been detected in human MoDCs [43]. On these considerations, we hypothesise that the pro-phagocytic effect of plasmin involves altered PDGF receptor signalling, but this requires further investigation.

Given that plasmin readily forms on diverse phagocytic targets (e.g. bacteria, tumour cells, amyloid, fibrin) and influences a variety of dendritic cell sub-types, these findings may have broader implications. This broad acting plasmin-mediated clearance mechanism denotes that NCC-aggregates formed as a consequence of cell necrosis represent a *bona fide* DAMP that binds/activates humoral factors and initiates favourable host responses to sterile tissue injury. The importance of plasmin to the modulation of dendritic cell function should now be assessed in disease settings such as burns and cancer – instances where immunosuppression [44] and substantial binding of t-PA/plasminogen to necrotic tissue [45] are commonplace.

## Supporting Information

S1 Fig Plasmin generation alone does not alter secretion of the immunomodulatory cytokines, IL-10 or IL-12, but decreases surface expression of multiple immune receptors. (A and B) MoDCs were incubated in the absence/presence of 1 nM t-PA + 100 nM plasminogen or 500 ng/mL LPS. After 24 h, the concentration of IL-10 (Panel A) and IL-12 (Panel B) was assessed in the conditioned media. Data are shown as mean ± s.e.m. (n = 3 independent experiments). *p<0.05 by 1-way ANOVA with Bonferroni post-hoc correction. (C-F) MoDCs were incubated in the absence/presence of 1 nM t-PA + 100 nM plasminogen. After 24 h, the cell-surface expression of CD70 (Panel C), CD80 (Panel D), CD274 (Panel E) and HLA-DR (Panel F) was assessed via flow cytometry. The mean fluorescence intensity of the immunosignal was normalized to that of untreated MoDCs. Data are displayed as mean ± s.e.m (n = 3 independent experiments). *p<0.05 by 2-tailed Welch’s unequal variances t-test.
(TIF)

S2 Fig Plasmin-treatment of MoDCs results in morphological changes but no cleavage of Annexin A2. (A) MoDCs treated with 0.1 nM plasmin, 1 nM plasmin (or 100 nM; not shown) for 24 h undergo drastic changes in cellular morphology as observed by phase-contrast microscopy. Scale bar is 50 μm. The depicted micrographs are representative of results obtained from 3 independent experiments. (B) MoDCs were treated with 100 nM plasmin, 100 nM inactive plasmin or 500 ng/mL LPS for 30 min. Total cell protein lysates were then prepared in the presence or absence of protease inhibitors and subjected to immunoblot analysis for Annexin A2. Plasmin-mediated Annexin A2 cleavage, as reported by others [26], was only observed when cell protein lysates were prepared in the absence of protease inhibitors. The depicted immunoblot is representative of results obtained from 3–6 independent experiments.
(TIF)

S3 Fig Plasmin increases the phagocytic capacity of mouse conventional dendritic cells. Bone marrow-derived mouse dendritic cells (BM-mDCs) were treated with 500 nm fluorescent microparticles in the presence/absence of 100 nM mouse plasmin. After 6 h, cells were stained with fluorophore-conjugated anti-CD11c,-CD11b and-Gr-1 antibodies and subjected to flow cytometry to determine the relative extent of microparticle uptake. BM-mDCs were gated as CD11c^positive^, CD11b^positive^, Gr-1^negative^ conventional dendritic cells. Data are shown as mean ± s.e.m. (n = 9 independent experiments). **p<0.01 by unpaired two-tail Student’s t-test.
(TIF)

S4 Fig Plasmin does not induce MoDC maturation. MoDCs were incubated in the presence/absence of 1 nM t-PA + 100 nM plasminogen. 24 h later, the cell surface expression of CD86 was assessed by flow cytometry. Comparable results were obtained across n = 3 independent experiments. Shown is a representative histogram of n = 1 experiment. CD86 expression of untreated (white peak; count = 8575) and t-PA + plasminogen treated (grey peak; count = - 7174). This histogram is an alternate depiction of data used in Fig 3C.
(TIF)

S5 Fig Original PRISM files used for statistical analysis. Raw data files of figures presented in this study. Files are presented in GraphPad Prism v.6.01 format.
(ZIP)

S1 Table Full Kinex comparison report of untreated MoDCs versus plasmin treated MoDCs at 3 hours. Significantly altered 'short-listed' proteins are highlighted where Pink represents up-regulated phospho-proteins; Blue represents down-regulated phospho-proteins. All highlighted 'short-listed' proteins were subjected to Ingenuity Pathway Analysis and to NCI-Pathway Interaction Database analysis (see Fig 5).
(XLSX)
