# Supplementary figures and images for: Dendritic Cell-Mediated Phagocytosis but Not Immune Activation Is Enhanced by Plasmin
Source: PLoS One. 2015 Jul 1;10(7):e0131216. doi: 10.1371/journal.pone.0131216 (PMC4488505; doi:10.1371/journal.pone.0131216)

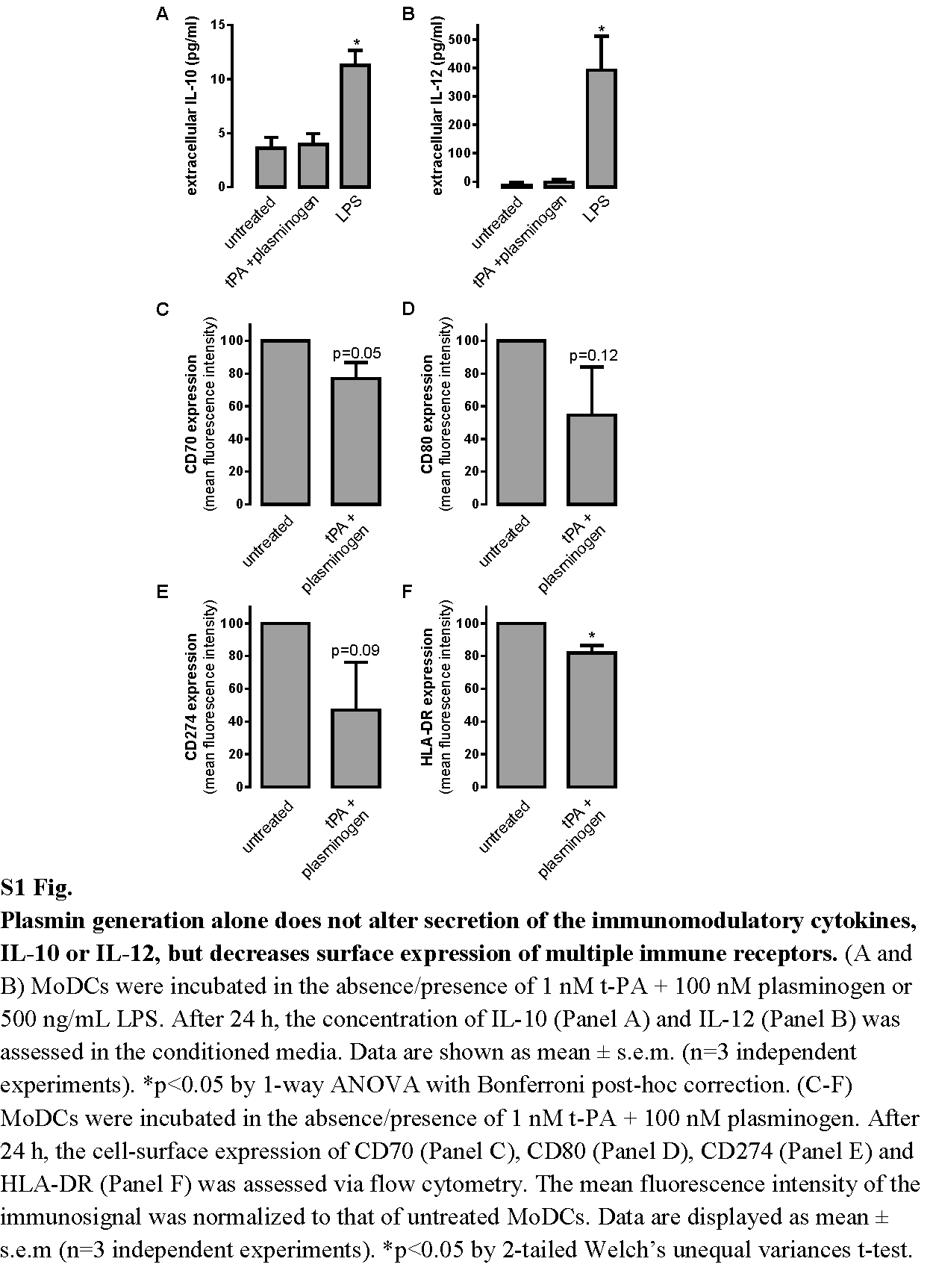

Supplement: S1 Fig — (A and B) MoDCs were incubated in the absence/presence of 1 nM t-PA + 100 nM plasminogen or 500 ng/mL LPS. After 24 h, the concentration of IL-10 (Panel A) and IL-12 (Panel B) was assessed in the conditioned media. Data are shown as mean ± s.e.m. (n = 3 independent experiments). *p<0.05 by 1-way ANOVA with Bonferroni post-hoc correction. (C-F) MoDCs were incubated in the absence/presence of 1 nM t-PA + 100 nM plasminogen. After 24 h, the cell-surface expression of CD70 (Panel C), CD80 (Panel D), CD274 (Panel E) and HLA-DR (Panel F) was assessed via flow cytometry. The mean fluorescence intensity of the immunosignal was normalized to that of untreated MoDCs. Data are displayed as mean ± s.e.m (n = 3 independent experiments). *p<0.05 by 2-tailed Welch’s unequal variances t-test. (TIF) [file pone.0131216.s001.tif]

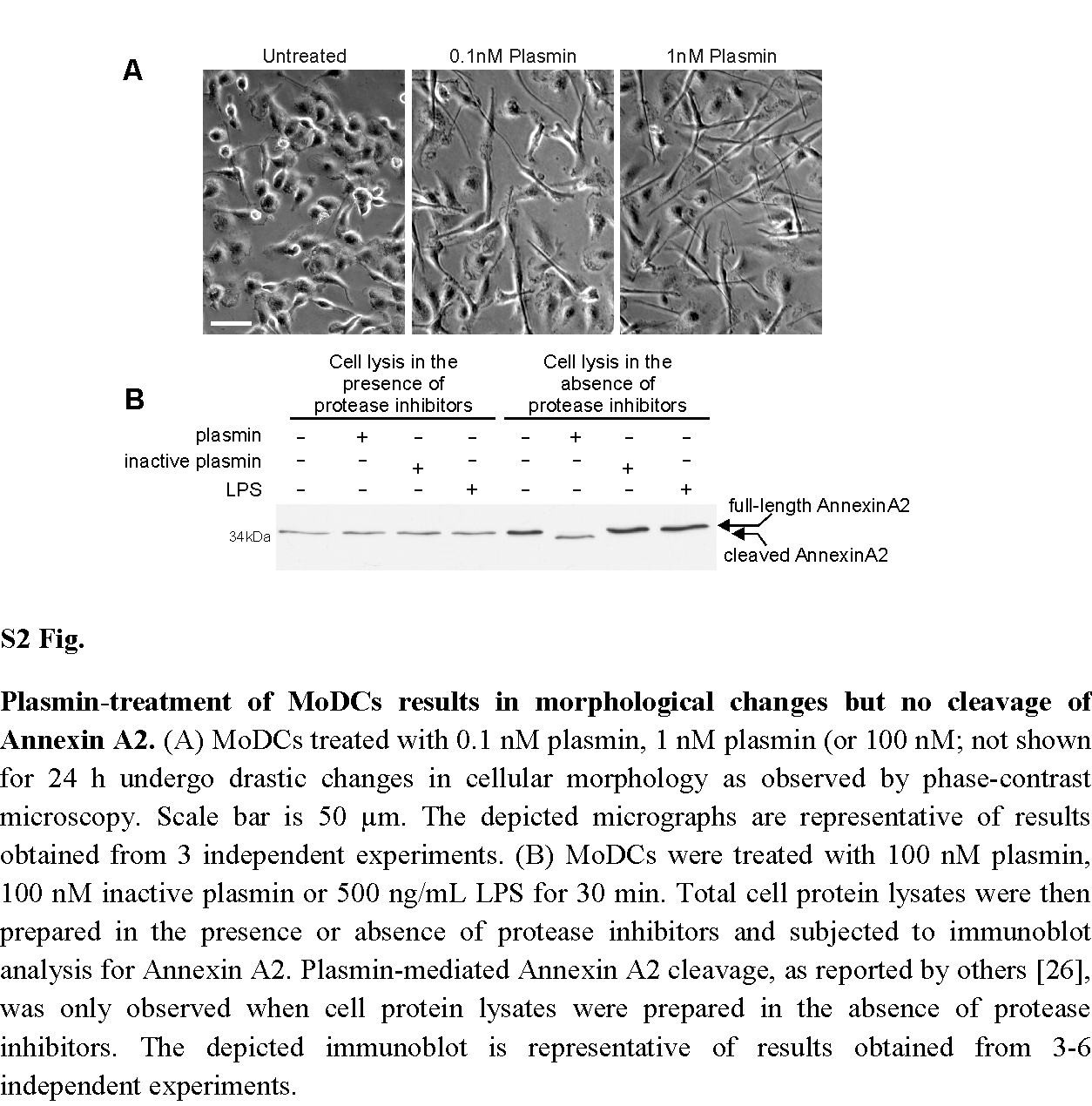

Supplement: S2 Fig — (A) MoDCs treated with 0.1 nM plasmin, 1 nM plasmin (or 100 nM; not shown) for 24 h undergo drastic changes in cellular morphology as observed by phase-contrast microscopy. Scale bar is 50 μm. The depicted micrographs are representative of results obtained from 3 independent experiments. (B) MoDCs were treated with 100 nM plasmin, 100 nM inactive plasmin or 500 ng/mL LPS for 30 min. Total cell protein lysates were then prepared in the presence or absence of protease inhibitors and subjected to immunoblot analysis for Annexin A2. Plasmin-mediated Annexin A2 cleavage, as reported by others [26], was only observed when cell protein lysates were prepared in the absence of protease inhibitors. The depicted immunoblot is representative of results obtained from 3–6 independent experiments. (TIF) [file pone.0131216.s002.tif]

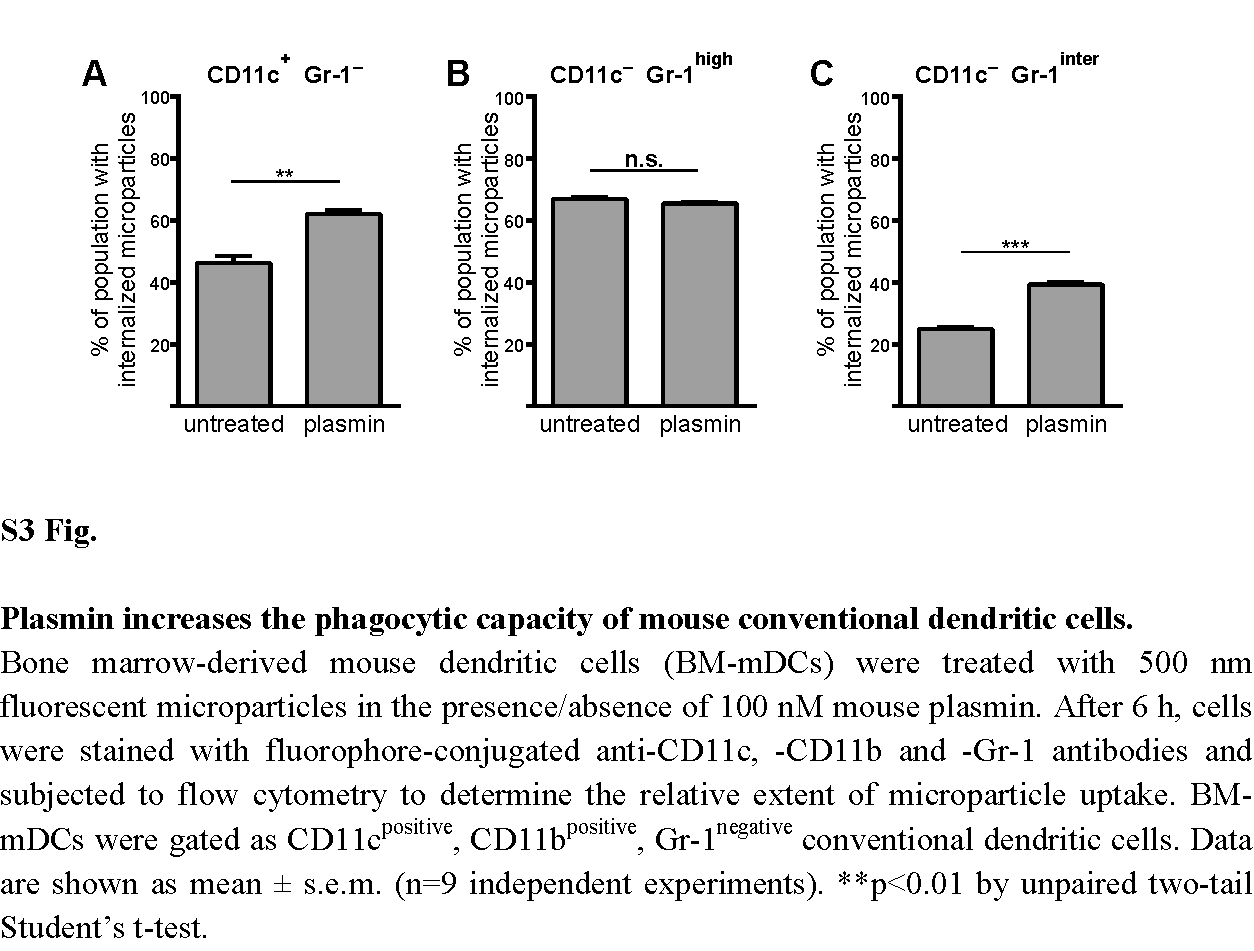

Supplement: S3 Fig — Bone marrow-derived mouse dendritic cells (BM-mDCs) were treated with 500 nm fluorescent microparticles in the presence/absence of 100 nM mouse plasmin. After 6 h, cells were stained with fluorophore-conjugated anti-CD11c,-CD11b and-Gr-1 antibodies and subjected to flow cytometry to determine the relative extent of microparticle uptake. BM-mDCs were gated as CD11cpositive, CD11bpositive, Gr-1negative conventional dendritic cells. Data are shown as mean ± s.e.m. (n = 9 independent experiments). **p<0.01 by unpaired two-tail Student’s t-test. (TIF) [file pone.0131216.s003.tif]

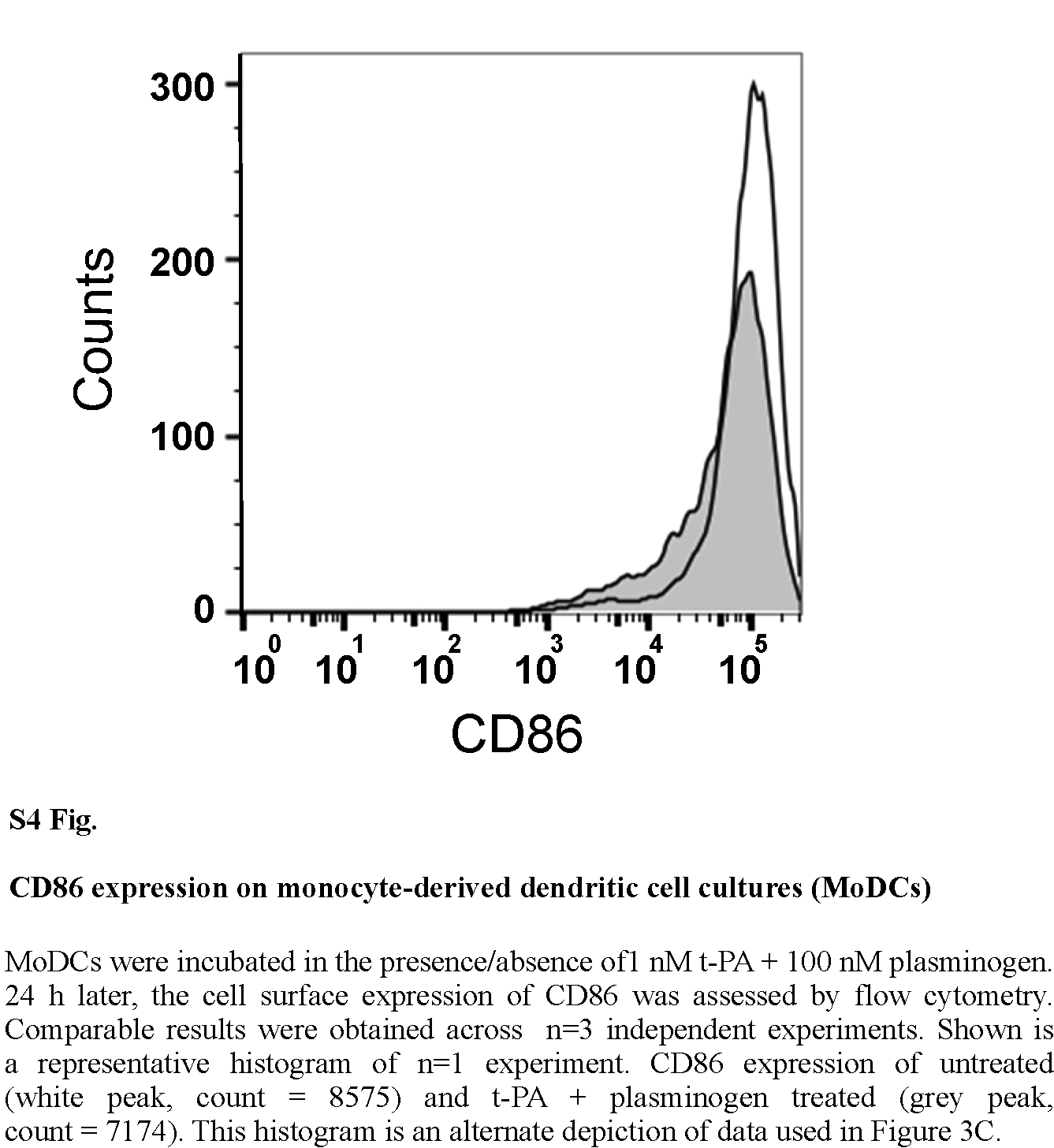

Supplement: S4 Fig — MoDCs were incubated in the presence/absence of 1 nM t-PA + 100 nM plasminogen. 24 h later, the cell surface expression of CD86 was assessed by flow cytometry. Comparable results were obtained across n = 3 independent experiments. Shown is a representative histogram of n = 1 experiment. CD86 expression of untreated (white peak; count = 8575) and t-PA + plasminogen treated (grey peak; count = - 7174). This histogram is an alternate depiction of data used in Fig 3C. (TIF) [file pone.0131216.s004.tif]
